# Leveraging longitudinal ACR CT Dose Index Registry data to improve interpretation of institutional CT dose performance

**DOI:** 10.1002/acm2.70896

**Published:** 2026-11-14

**Authors:** Azmul H. Siddique, Mike Weaver, Gary Ge, Leslie Anaskevich, Jie Zhang

**Affiliations:** ^1^ Department of Radiology University of Kentucky Lexington Kentucky USA

**Keywords:** ACR, CT, CT dose, diagnostic reference level, DIR, Dose Index Registry, dose performance, dose reference level, longitudinal, NRDR

## Abstract

**Background:**

The American College of Radiology CT Dose Index Registry (DIR) is a national benchmarking program that enables institutions to compare CT dose metrics with aggregated reference distributions to support CT radiation dose optimization and quality assurance. In practice, benchmarking is typically reviewed at discrete reporting intervals, which can be limited by benchmark shifts, transient variation, or incomplete reference data.

**Purpose:**

This study evaluates how longitudinal DIR analysis improves interpretation of institutional CT dose performance and supports protocol optimizations.

**Methods:**

Longitudinal DIR data from 2020 to 2025 were analyzed for 10 common CT exams across 21 reporting quarters for both adult and pediatric. CTDI_vol_, DLP, and SSDE distributions (25th, 50th, 75th percentiles) were compared with exam‐specific DIR reference percentiles. Exams with *n* ≥ 30 per reporting interval were included for analysis. Exceedance rates, defined as the median value of facility metric exceeding the DIR 75th percentile (Diagnostic Reference Level [DRL]), were evaluated across exams and reporting intervals. Linear mixed‐effects (LME) regression was used to evaluate longitudinal trends and identify exams demonstrating statistically significant dose drift. Coefficient of variation (CV) and F‐test were performed to assess year‐to‐year variability.

**Results:**

Four of ten adult exams remained within DRL across all intervals for both CTDI_vol_ and DLP. CT Neck with IV contrast exceeded the CTDI_vol_ and DLP DRLs in 19/21 intervals, and CT C‐spine without IV contrast exceeded the DLP DRL in 19/21 intervals. Pediatric data demonstrated exceedance rates greater than 50% for both CTDI_vol_ and DLP in those same exams. For adult exams with seven available DIR SSDE benchmarks, five remained within the SSDE DRL across all intervals. LME analysis identified a significant increase in institutional dose metrics in 2025 (*β*_2_ = 0.139; *p* = 0.001), with CT C‐Spine contributing largest positive shift (30.56%) for adults. Facility median variability (CV) ranged from 4.6%–127%, whereas DIR 75th percentile variability ranged from 2.8% to 21.9%.

**Conclusion:**

Longitudinal analysis of ACR DIR executive summary data extends isolated quarterly review by contextualizing exceedances over time, accounting for benchmark variability, and distinguishing persistent exam behavior from transient effects. This supports improved interpretation of institutional CT dose performance and targeted exam triage.

## 1 INTRODUCTION

The American College of Radiology (ACR) CT Dose Index Registry (DIR) is a national dose benchmarking program designed to support quality improvement and radiation dose optimization in computed tomography (CT). Established in 2011 as part of the ACR National Radiology Data Registry (NRDR), the DIR enables participating institutions to submit de‐identified CT dose data and compare their performance against aggregated national reference distributions for common CT examinations.^1^ The registry was developed to address variability in CT radiation dose across institutions by providing a standardized framework for peer comparison and quality assurance.^1^, ^2^, ^3^, ^4^ It has since become an important tool for assessing institutional dose performance and guiding protocol optimization.

DIR feedback is primarily delivered through quarterly executive summary reports and benchmark tables (executive summary tables) that compare facility dose distributions with national DIR percentiles, including dose metrics such as volume computed tomography dose index (CTDI_vol_), dose‐length product (DLP), and size‐specific dose estimate (SSDE). The executive summary tables list the exams for the Top 10 RPID Groupname to facilitate comparison with all DIR facilities.^5^ Diagnostic reference levels (DRLs), commonly defined using the DIR 75th percentile for a given examination and dose metric, provide a reference for identifying potentially elevated dose performance.^1^, ^2^ Additional visual tools, such as high‐volume boxplots, highlight performance for commonly reported examinations within each reporting period.^6^ Together, these measures allow facilities to evaluate scanner output, examination protocols, and patient‐size considerations within a unified benchmarking framework.^7^, ^8^, ^9^

In practice, DIR data are typically reviewed at discrete reporting intervals with emphasis on whether an exam exceeds DRL in a single quarter. Interpretation based on isolated intervals can be misleading. Apparent exceedances may reflect transient fluctuations in case mix, operational factors, or changes in national reference distributions rather than true exam‐level dose issues. Conversely, gradual changes in dose metrics may remain undetected when values remain just below benchmark thresholds. Temporal variability in CT dose metrics and incomplete benchmark availability across exams and patient populations, particularly for pediatric examinations and SSDE, further complicate interpretation based on isolated quarterly review.^4^, ^10^

Longitudinal analysis of DIR quarterly benchmark data across multiple consecutive reporting intervals may provide a more informative framework for interpreting exam‐level CT dose performance. Tracking benchmark movement, intermittent exceedance, and intervals with incomplete reference data over time may reveal patterns that could be missed using isolated quarterly review alone. Using multi‐year adult and pediatric DIR data, this study aims to demonstrate how longitudinal visualization and statistical analysis can enhance CT dose management and exam triage through evaluation of exceedance patterns across CTDI_vol_, DLP, and SSDE.

## 2 METHODS

### 2.1 Data sources and selection

CT dose benchmarking data from 2020 to 2025 (study period) were obtained from quarterly ACR CT DIR executive summary reports for the 10 most common adult and 10 most common pediatric examinations (Table 1). For each reporting interval, executive summaries provided facility‐specific and DIR benchmark 25th, 50th (median), and 75th percentile values for CTDI_vol_, DLP, and SSDE, along with corresponding exam counts. These data were extracted for longitudinal analysis. Although data for this study were compiled from quarterly executive summary reports, longitudinal dose index data are also available to registered DIR users through the ACR CT Administrative Aggregate interactive report, which may facilitate application of this longitudinal approach without requiring compilation of individual quarterly reports.^11^ Two reporting quarters were available in 2020 and three in 2025, with complete quarterly coverage for all intervening years, resulting in a total of 21 quarters evaluated per exam. Consistent with ACR DIR reporting practices, national benchmark data were available only for examinations meeting minimum registry participation thresholds (≥10 000 adult examinations and ≥2000 pediatric examinations across participating institutions).^11^, ^12^ Pediatric data were available in five DIR‐defined age groups: 00–02, 03–06, 07–10, 11–14, and 15–18 years. Each examination‐age group combination (exam‐age group) was analyzed independently. For the present study, facility data were included in the longitudinal analysis only if at least 30 examinations were available within a reporting interval and data were available for a minimum of three quarters throughout the study period. These thresholds were chosen to mitigate the effects of small‐sample variability and provide more robust facility‐level performance estimates.

**TABLE 1:** Top 10 common exams listed for the adult and pediatric group.

Adult	Pediatric
CT Abdomen Pelvis Kidney WO IVCON	CT Lower Extremity (LE) WO IVCON
CT Abdomen Pelvis W IVCON	CT Abdomen Pelvis W IVCON
CT Abdomen Pelvis WO IVCON	CT Abdomen Pelvis WO IVCON
CT Cervical Spine (C‐Spine) WO IVCON	CT Cervical Spine WO IVCON
CT Chest Abdomen Pelvis W IVCON	CT Chest Abdomen Pelvis W IVCON
CT Chest Pulmonary Arteries W IVCON	CT Head Paranasal Sinuses WO IVCON
CT Chest W IVCON	CT Chest W IVCON
CT Chest WO IVCON	CT Chest WO IVCON
CT Head Brain WO IVCON	CT Head Brain WO IVCON
CT Neck W IVCON	CT Neck W IVCON

*Note*: Pediatric data were further stratified into the following age groups: 00–02, 03–06, 07–10, 11–14, and 15–18.

Abbreviations: IVCON, intravenous contrast; W, with; WO, without.

### 2.2 Longitudinal data analysis

For each adult exam, pediatric exam‐age group, and dose metric combination meeting the inclusion criteria, DRL exceedance rate was calculated as the percentage of reporting intervals during the study period in which the facility median exceeded the corresponding DIR 75th percentile. Exams were subsequently categorized according to the frequency and persistence of exceedance across the 21‐quarter study period. To complement quantitative exceedance rates, qualitative longitudinal visualization was performed for each exam by plotting facility percentile distributions over time, overlaid with DIR benchmark percentiles and their associated ± 1 standard deviation bands. To generate these standard deviation bands, the DIR 25th, 50th, and 75th percentile values reported across all quarters were compiled separately for each exam and dose metric, and one standard deviation was calculated for each percentile. These standard deviation bands represent the absolute temporal variability of each DIR percentile across reporting intervals over the entire study period and provide visual context regarding the variability of the DIR benchmarks against which facility performance was compared. Qualitative observations were used to interpret quantitative exceedance findings and to distinguish sustained protocol behavior from transient or potential data anomalies.

### 2.3 Statistical analysis

To achieve better exam‐level optimization triage, longitudinal changes in dose performance were further evaluated using linear mixed effects (LME) regression. It was restricted to datasets with complete quarterly reporting across the entire study period to ensure stable parameter estimation and thus, exams with less than 21 quarters of available data were excluded. Based on these criteria, the evaluation was limited to adult CTDI_vol_ data only. This analysis was performed to demonstrate the application of LME regression for prioritizing exams demonstrating recent dose deviation from their historical longitudinal performance.

Median dose values were log‐transformed prior to modeling to evaluate relative changes over time and to stabilize variance across exams with differing baseline dose magnitudes. An initial LME regression model incorporating time as a continuous variable was used to estimate overall longitudinal drift across reporting intervals. To identify exams demonstrating recent upward deviation beyond the historical trend, the model was extended to include an indicator variable representing the most recent calendar year (e.g., 2025), $I_{2025,\mathrm{ij}}$. It is important to note that all quarterly observations were retained in the model, and the annual indicator did not replace the quarterly temporal structure. Rather, the calendar year interval was selected as a clinically practical assessment period aligned with annual CT protocol review. The 2025 indicator therefore quantified whether dose performance during the most recent annual review period deviated from the historical quarterly trajectory, allowing exams to be prioritized for further investigation and optimization.

For the selected dose metric, the following model was fit:

(1) $$\log\left(Y_{ij}\right)=\beta_{0}+\beta_{1}t_{\mathrm{ij}}+\beta_{2}I_{2025,\mathrm{ij}}+b_{0i}+b_{1i}t_{\mathrm{ij}}+b_{2i}I_{2025,\mathrm{ij}}+\varepsilon_{ij}$$

where:

$Y_{ij}$ represents the facility median dose value for exam 𝑖 at reporting interval 𝑗;

$t_{ij}$ denotes the continuous time index (quarter number);

$I_{2025,\mathrm{ij}}$ is an indicator variable equal to 1 for reporting intervals in calendar year 2025 and 0 otherwise;

$\beta_{0}$ is the fixed‐effect intercept;

$\beta_{1}$ represents average longitudinal drift (slope) per reporting interval across all years;

$\beta_{2}$ represents the deviation in 2025 relative to the fitted longitudinal trend;

$b_{0i}$, $\;b_{1i}$, $b_{2i}$ are exam‐specific random effects for intercept, long‐term drift, and 2025 deviation, respectively;

$\varepsilon_{ij}∼N\left(0,\sigma^{2}\right)$ represents residual error.

Model parameters (i.e., $\beta_{0}$, $\;b_{1i}$, etc.) were estimated using restricted maximum likelihood (REML). The exam‐specific long‐term drift per reporting interval was defined as $\beta_{1}+b_{1i}$, and the exam‐specific deviation during 2025 was defined as $\beta_{2}+b_{2i}$. Because the outcome was log‐transformed, model coefficients were interpreted as multiplicative effects and converted to percent change in 2025 using:

(2) $$\%\Delta_{2025,i}=100\left(e^{\beta_{2}+b_{2i}}-1\right)$$

Statistical significance of the fixed‐effect slope (i.e., $\beta_{1}$) and the 2025 deviation (i.e., $\beta_{2}$) were assessed using two‐sided hypothesis testing with a significance level of *α* = 0.05. Exam‐specific random‐effect estimates were extracted from the fitted model and used to rank exams according to the magnitude of their estimated 2025 deviation from trend. These exam‐specific estimates represent conditional best linear unbiased predictions (BLUPs) rather than independently tested fixed effects.

Dose variability was further quantified using the coefficient of variation (CV), calculated separately from quarterly facility median values and quarterly DRL values within each calendar year and across the entire study period. Facility median and DRL (DIR 75th percentile) values were selected to maintain consistency with the primary exceedance analysis and to characterize the temporal stability of both quantities used in this benchmark comparison. The overall CV represents the same absolute temporal variability depicted by its standard deviation band, normalized to the mean to provide a unitless measure of relative variability. Annual CV values similarly quantified relative variability across quarterly observations within each calendar year. Year‐to‐year differences in variability were assessed using F‐tests for equality of variances (*α* = 0.05). To ensure stable variance estimation, F‐tests were performed only for year pairs in which both years contained a minimum of four quarterly observations. Given the limited number of observations within each year (*n* = 4), these tests were considered exploratory and were used to complement the quantitative CV analysis. For each exam, the variance of CTDI_vol_ ​values in year y was compared with that of the preceding year y‐1 (2022 vs. 2021, 2023 vs. 2022, and 2024 vs. 2023).

All data and statistical analysis were performed using Python (Python Software Foundation, version 3.12.3). ChatGPT (GPT‐5.6 Sol, OpenAI) was used for grammar, spelling, and stylistic editing of the manuscript. All AI‐assisted edits were reviewed and approved by the authors.

## 3 RESULTS

### 3.1 Adult longitudinal dose performance

Table 2 shows the exceedance rates of top 10 common CT adult exams for CTDI_vol_, DLP, and SSDE throughout the entire study period. Across 21 reporting intervals, CTDI_vol_ exceedance rates ranged from 0% to 90.48%, with two exams exceeding 50%: CT Neck W IVCON (19/21, 90.48%) and CT C‐Spine WO IVCON (11/21, 52.38%). Four exams, CT Abdomen Pelvis Kidney WO IVCON, CT Abdomen Pelvis W IVCON, CT Chest Abdomen Pelvis W IVCON, and CT Chest WO IVCON, were within the CTDI_vol_ DIR threshold consistently.

**TABLE 2:** Facility exceedance rates of 10 most common adult CT exams for CTDI_vol_, DLP, and SSDE.

Exams	CTDI_vol_ exceedance rate (%)	DLP exceedance rate (%)	SSDE exceedance rate (%)
**CT ABDOMEN PELVIS KIDNEY WO IVCON**	0.00% (0/21)	0.00% (0/21)	0.00% (0/21)
**CT ABDOMEN PELVIS W IVCON**	4.76% (1/21)	9.52% (2/21)	0.00% (0/21)
**CT ABDOMEN PELVIS WO IVCON**	0.00% (0/21)	0.00% (0/21)	0.00% (0/21)
**CT C‐SPINE WO IVCON**	52.38% (11/21)	90.48% (19/21)	N/A
**CT CHEST ABDOMEN PELVIS W IVCON**	0.00% (0/21)	0.00% (0/21)	5.26% (1/19)
**CT CHEST PULMONARY ARTERIES W IVCON**	33.33% (7/21)	71.43% (15/21)	9.52% (2/21)
**CT CHEST W IVCON**	9.52% (2/21)	80.95% (17/21)	0.00% (0/21)
**CT CHEST WO IVCON**	0.00% (0/21)	0.00% (0/21)	0.00% (0/21)
**CT HEAD BRAIN WO IVCON**	28.57% (6/21)	38.10% (8/21)	N/A
**CT NECK W IVCON**	90.48% (19/21)	90.48% (19/21)	N/A

*Note*: Cells with “N/A” did not meet the inclusion criteria set for this study. Values in parentheses represent the number of quarters exceeding DIR benchmark out of the total number of quarters evaluated.

DLP exceedance rates were generally higher than those observed for CTDI_vol_. Four adult exams demonstrated exceedance rates greater than 50%: CT Neck W IVCON and CT C‐Spine WO IVCON (19/21, 90.48%), CT Chest Pulmonary Arteries W IVCON (15/21, 71.43%), and CT Chest W IVCON (17/21, 80.95%). The same four exams that remained below the CTDI_vol_ DIR threshold also did not exceed the DLP DIR threshold in any reporting interval.

SSDE exceedance events were infrequent with only two exams demonstrating exceedance rates: CT Chest Abdomen Pelvis W IVCON (5.26%, 1/19) and CT Chest Pulmonary Arteries W IVCON (9.52%, 2/21). Five exams, as demonstrated in Table 2, did not have any SSDE exceedance across all available reporting intervals.

The plots for longitudinal data showed various temporal patterns. Figure 1 shows a pattern for intermittent exceedance and temporal variability within the CT C‐Spine WO IVCON exam. Facility median CTDI_vol_ values increased progressively beginning in early 2021, then exceeded the DIR threshold through 2024 and remained consistently above the threshold in 2025. During 2022–2023, CTDI_vol_ values remained near the DIR threshold while DLP values exceeded the threshold across consecutive reporting intervals. In the final three reporting quarters of 2025, both CTDI_vol_ and DLP values increased substantially compared to prior reporting periods.

**FIGURE 1:**
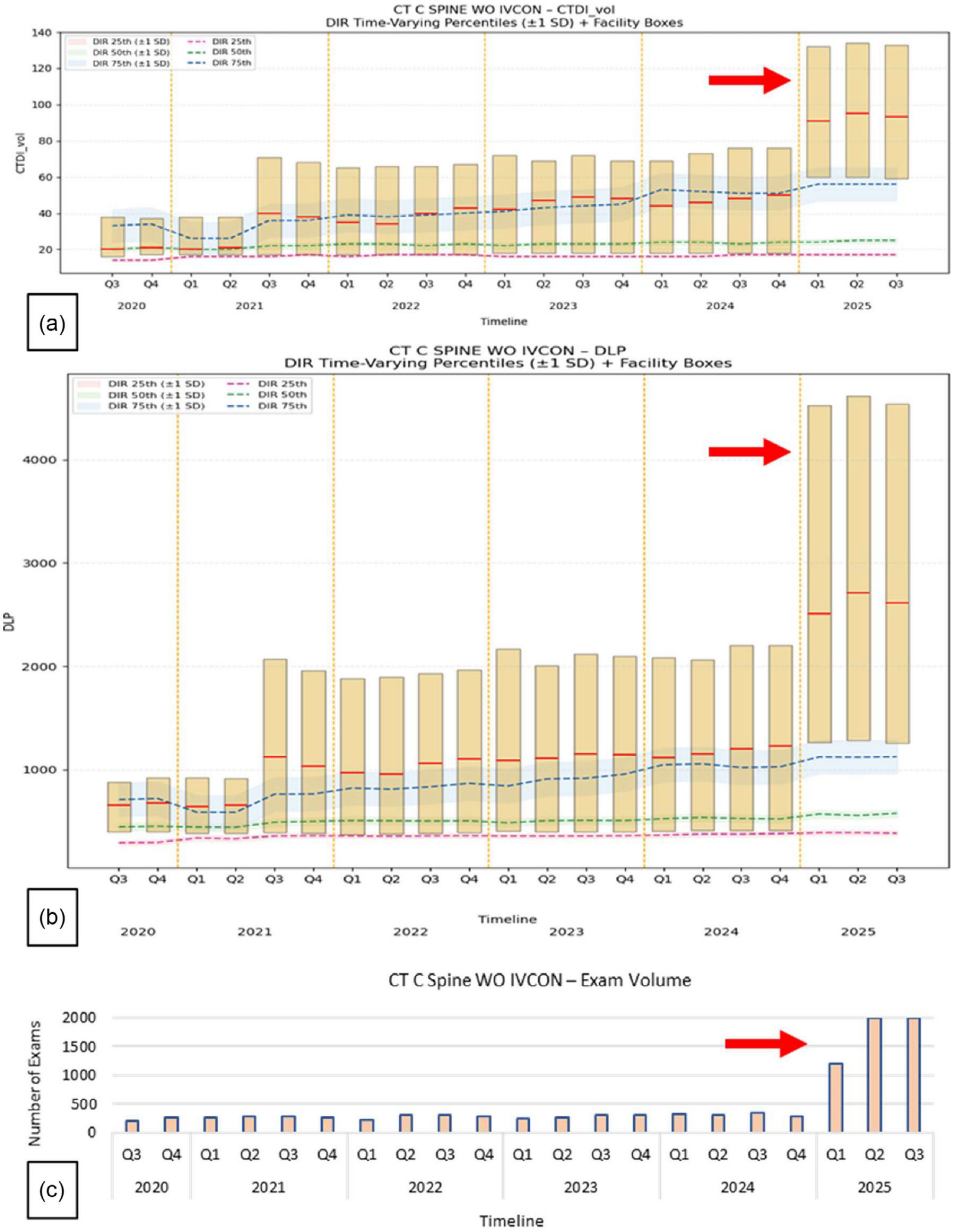
Adult CT C‐Spine WO IVCON CTDI_vol_ (a), DLP (b) longitudinal analysis, and (c) exam volume plots. CTDI_vol_ began increasing in early 2021 and intermittently exceeded the DIR threshold through 2024 before remaining consistently elevated in 2025 (red arrow). DLP exceeded the threshold across multiple intervals during 2022–2023 and increased substantially during the final reporting quarters of 2025 (red arrow). Exam volume increased markedly during 2025 (red arrow).

Figure 2 shows a different temporal pattern observed in CT Chest Pulmonary Arteries W IVCON exam, particularly related to benchmark drift, where facility median CTDI_vol_ values remained relatively stable across reporting intervals. However, the DIR 75th percentile threshold decreased between 2023 and 2024, resulting in exceedance during subsequent quarters despite minimal change in the facility median trajectory. In contrast, CT Head Brain WO IVCON exams (Figure 3a,b) demonstrated a consistent gradual upward trajectory in facility median CTDI_vol_ and DLP. Values approached the DIR threshold in 2023 and exceeded the threshold in 2024 and 2025, while DIR benchmark percentiles remained relatively stable over the same period.

**FIGURE 2:**
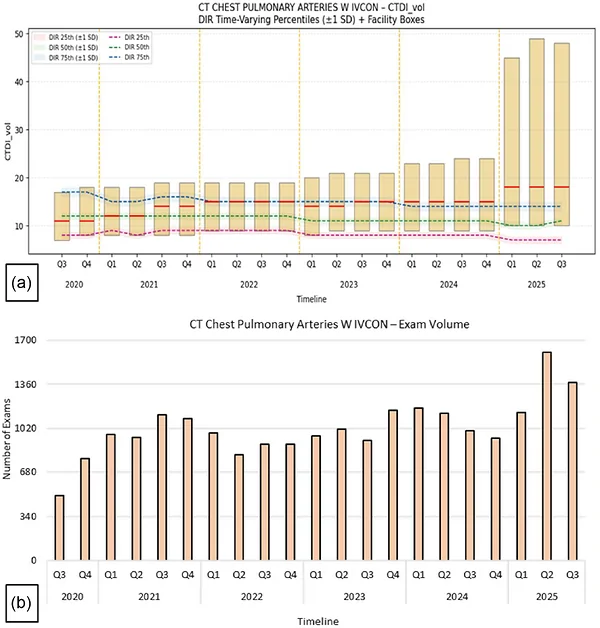
Adult CT Chest Pulmonary Arteries W IVCON CTDI_vol_ (a) and exam volume (b) longitudinal analyses. This example illustrates how facility median values can remain relatively stable while changes in the DIR threshold over time (between 2023 and 2024) result in protocol exceedance.

**FIGURE 3:**
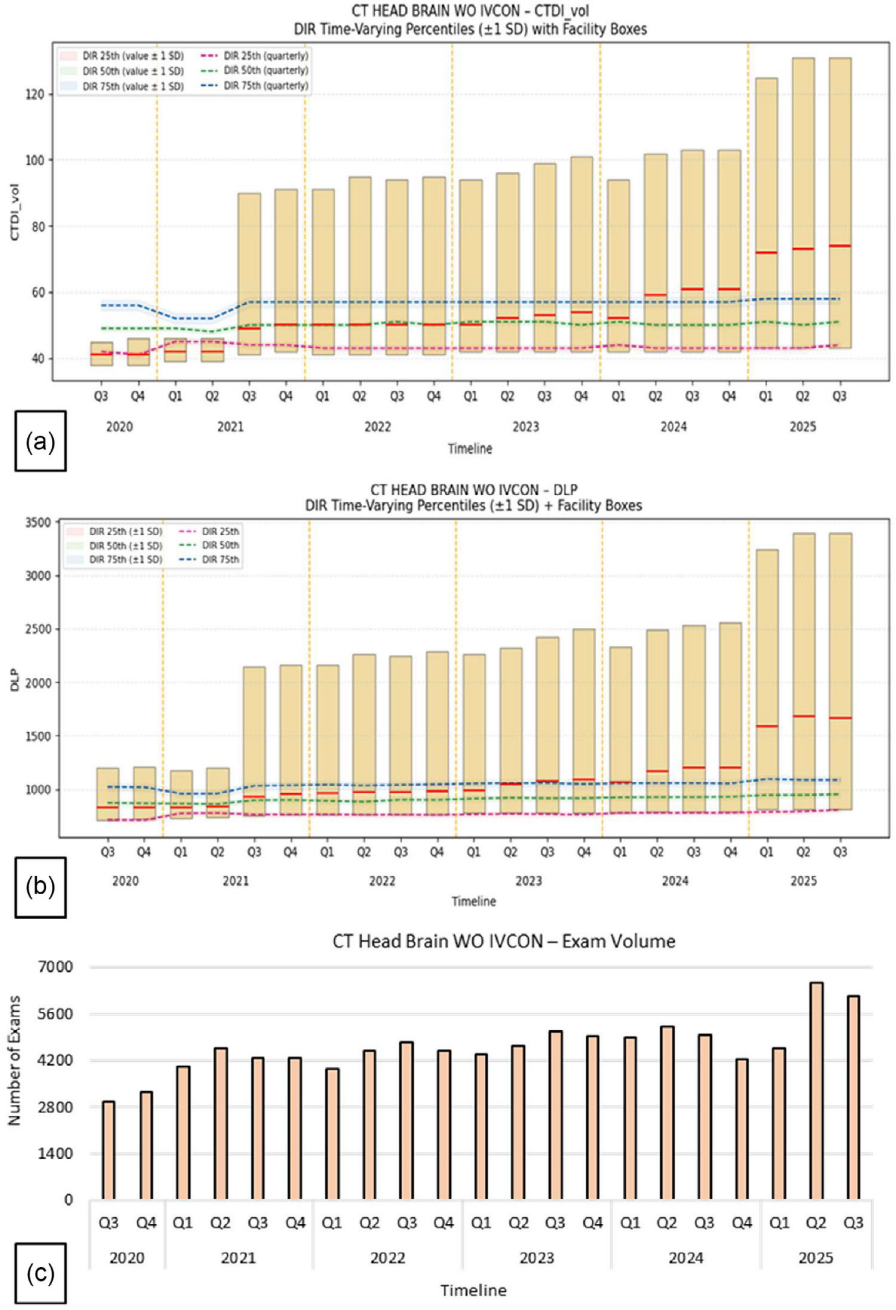
Adult CT Head Brain WO IVCON CTDI_vol_ (a), DLP (b), and exam volume (c) longitudinal analysis plots. Facility median CTDI_vol_ and DLP values demonstrated a gradual upward trend across reporting intervals, approaching the DIR threshold in 2023 and exceeding it during 2024 and 2025, while DIR benchmark percentiles remained relatively stable.

In addition, CT Neck W IVCON (Figure 4) exhibited exam‐specific behavior, where persistent elevation was observed. Facility median CTDI_vol_ values exceeded the DIR threshold beginning mid‐2021 and remained elevated across all subsequent reporting intervals. Corresponding DLP values demonstrated sustained exceedance across the same intervals as shown in Table 2.

**FIGURE 4:**
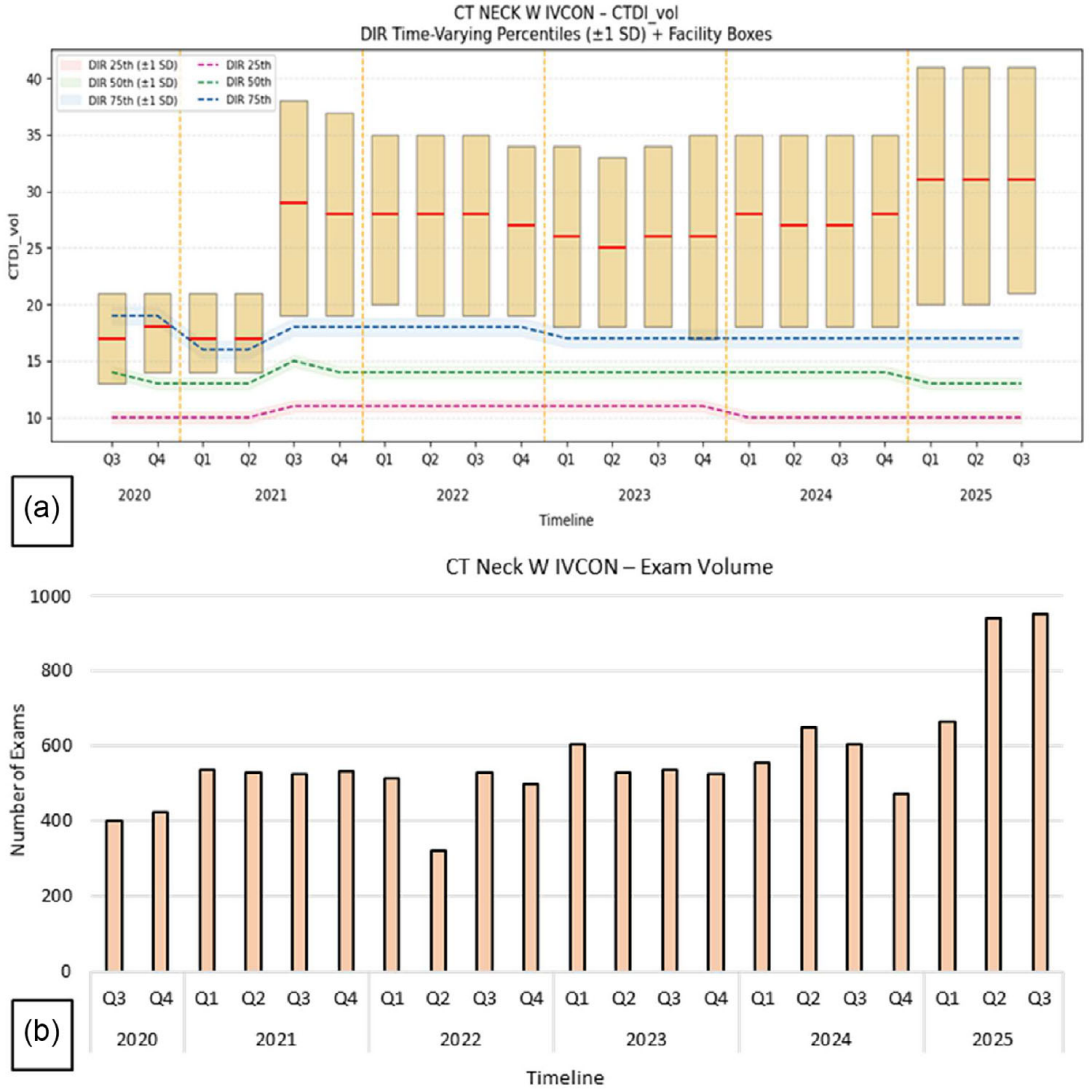
Adult CT Neck W IVCON CTDI_vol_ (a) and exam volume (b) longitudinal analysis plots. Facility median CTDI_vol_ exceeded the DIR threshold beginning in 2021 and remained elevated across nearly all subsequent reporting intervals.

### 3.2 Pediatric longitudinal dose performance

For pediatrics, CTDI_vol_ exceedance rates greater than 50% were observed in two exam‐age groups: CT C‐Spine WO IVCON in the 0–2‐year age group (4/5, 80%) and CT Neck W IVCON in the 15–18‐year age group (3/3, 100%). As demonstrated in Table 3, four exams had moderate exceedance rates—CT Abdomen Pelvis W IVCON (15–18 age group, 20%), CT C‐Spine WO IVCON (15–18 age group, 33.33%), and CT Head Brain WO IVCON (00–02 age group, 30% and 15–18 age group, 14.29%). 5/11 exam‐age groups had no CTDI_vol_ exceedance across all available reporting intervals.

**TABLE 3:** Facility exceedance rates of 10 most common pediatric CT exams for CTDI_vol_, DLP, and SSDE.

Exams	Age group	CTDI_vol_ exceedance rate (%)	DLP exceedance rate (%)	SSDE exceedance rate (%)
**CT ABDOMEN PELVIS W IVCON**	15–18	20.00% (4/20)	5.00% (1/20)	7.14% (1/14)
**CT C‐SPINE WO IVCON**	00–02	80.00% (4/5)	100.00% (5/5)	N/A
	15–18	33.33% (2/6)	33.33% (2/6)	N/A
**CT CHEST W IVCON**	15–18	0.00% (0/5)	0.00% (0/5)	N/A
**CT CHEST WO IVCON**	15–18	0.00% (0/8)	0.00% (0/8)	N/A
**CT HEAD BRAIN WO IVCON**	00–02	30.00% (6/20)	30.00% (6/20)	N/A
	03–06	0.00% (0/14)	0.00% (0/14)	N/A
	07–10	0.00% (0/15)	0.00% (0/15)	N/A
	11–14	0.00% (0/16)	0.00% (0/15)	N/A
	15–18	14.29% (3/21)	9.52% (2/21)	N/A
**CT NECK W IVCON**	15–18	100.00% (3/3)	66.67% (2/3)	N/A

*Note*: Cells with “N/A” did not meet the inclusion criteria set for this study. Values in parentheses represent the number of quarters exceeding DIR benchmark out of the total number of quarters evaluated.

For DLP, exceedance behavior followed similar exam‐age group patterns, where CT C‐Spine WO IVCON in the 0–2‐year age group exceeded the DIR threshold in all evaluable reporting intervals (5/5, 100%) and CT Neck W IVCON in the 15–18‐year age group exceeded all but one (2/3, 66.67%). SSDE benchmark data were available for only one pediatric protocol‐age group combination: CT Abdomen Pelvis W IVCON in the 15–18‐year age group and exceeded the threshold in 1 of 14 available reporting intervals.

Longitudinal plots for pediatrics also demonstrated intermittent exceedance, particularly for CT Head Brain WO IVCON exams in the 15–18‐year age group, across reporting intervals (Figure 5a,b). Facility median CTDI_vol_ values remained below the DIR threshold from 2020 through 2023. An initial exceedance was observed in the first quarter of 2024, followed by return to values below the threshold for the remainder of that year. Exceedance recurred in the second and third quarters of 2025. Throughout the study period, DIR benchmark percentiles remained relatively stable. A similar temporal pattern was observed for DLP in the same exam‐age group combination (Figure 5b: Year 2024, Quarter 1 and Year 2025, Quarter 2 & 3).

**FIGURE 5:**
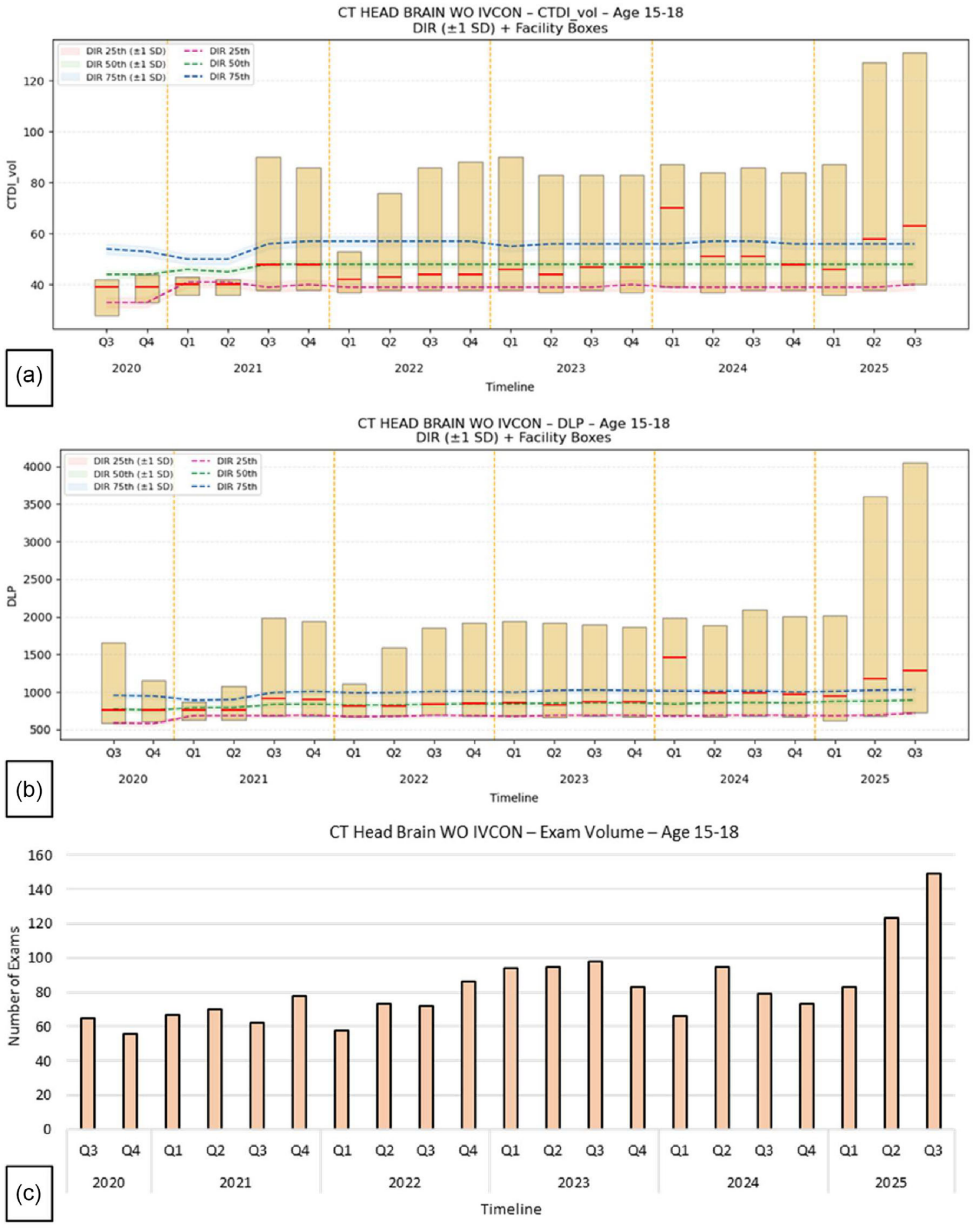
Pediatric CT Head Brain WO IVCON age 15–18 CTDI_vol_ (a), DLP (b), and exam volume (c) longitudinal analysis plots. Facility median CTDI_vol_ remained below the DIR threshold from 2020 to 2023, with intermittent exceedance observed in early 2024 and again during the second and third quarters of 2025. A similar pattern was observed for DLP, with exceedance occurring in the second and third quarters of 2025 and increased variability in later reporting intervals.

### 3.3 Statistical analysis

The adult CTDI_vol_ ​dataset included 10 exams with complete quarterly reporting from 2020 through 2025, yielding a total of 210 observations. All exams contributed equally to the LME regression model. The fitted model yielded a residual variance of $\sigma^{2}=0.0129$ on the log‐transformed scale. The fixed time effect was not statistically significant (*β*_1_ = 0.009 per quarter on the log scale; 95% CI: −0.074 to 0.093; *p* = 0.824), indicating no evidence of steady facility‐wide linear drift across the full study period. In contrast, the 2025 deviation term was statistically significant (*β*_2_ = 0.139; 95% CI: 0.060–0.219; *p* = 0.001). This corresponded to an estimated 14.9% increase in CTDI_vol_ ​during calendar year 2025 relative to historical performance. The observed 2025 increase in CTDI_vol_ was not uniform across all exams but varied in magnitude amongst them. Estimated exam‐specific 2025 shifts ranged from 4.26% to 30.56% relative to historical performance (Table 4). Ranking exams according to the magnitude of their estimated 2025 deviation identified CT C‐Spine WO IVCON as demonstrating the largest increase (30.56%), followed by CT Chest W IVCON (21.35%), CT Chest Abdomen Pelvis W IVCON (19.90%), and CT Head Brain WO IVCON (19.90%) (Table 4).

**TABLE 4:** Estimated exam‐specific 2025 CTDI_vol_ shifts derived from the linear mixed‐effects (LME) regression.

Exams	2025 shifts (%)
CT C‐Spine WO IVCON	30.56
CT Chest W IVCON	21.35
CT Chest Abdomen Pelvis W IVCON	19.90
CT Head Brain WO IVCON	19.90
CT Chest WO IVCON	16.41
CT Abdomen Pelvis WO IVCON	13.72
CT Chest Pulmonary Arteries W IVCON	11.45
CT Neck W IVCON	7.59
CT Abdomen Pelvis W IVCON	6.92
CT Abdomen Pelvis Kidney WO IVCON	4.26

*Note*: Values represent the total model‐predicted percent change in 2025 relative to historical performance, incorporating both the overall fixed 2025 effect and exam‐specific random‐effect deviations.

Year‐to‐year variance analysis for facility and DIR data showed statistically significant changes between consecutive reporting years (Table 5). For the facility data, overall CV ranged from 4.6% (adult CT Abdomen Pelvis Kidney WO IVCON) to as high as 127% (pediatric CT Head Brain WO IVCON, 0–2 age group) for the entire study period. Repeated variance changes were observed, such as in the adult CT Abdomen Pelvis W IVCON, where CV values ranged from 28.5% in 2021 to no variance in 2023, with multiple consecutive year‐to‐year comparisons reaching statistical significance. In contrast, several exams such as adult CT Abdomen Pelvis Kidney WO IVCON demonstrated consistently low variability across the study period, with annual CV values generally remaining below 10% and no significant year‐to‐year variance changes detected. Among all exams, pediatric CT Head Brain WO IVCON in the 0–2‐year age group demonstrated the largest year‐to‐year variability, with a peak CV of 61.3% in 2021.

**TABLE 5:** Annual CV results for CTDI_vol_ of facility median value percentile (represented as Facility) and DIR 75th percentile (represented as Diagnostic Reference Level, DRL). Higher CV values indicate greater variability in dose performance across quarterly reporting intervals. Color‐coded cells show F‐test results, where red indicates a statistically significant difference, and green indicates no significant difference between that year and the preceding year. F‐tests were performed only for year pairs in which both years contained a minimum of four quarterly observations (i.e., 2022 vs. 2021, 2023 vs. 2022, and 2024 vs. 2023).

		2020		2021		2022		2023		2024		2025		Overall	
Exams		Facility	DRL	Facility	DRL	Facility	DRL	Facility	DRL	Facility	DRL	Facility	DRL	Facility	DRL
Adult	1	7.4%	0.0%	4.9%	3.5%	6.1%	3.7%	0.0%	3.4%	0.0%	3.5%	6.0%	0.0%	4.6%	8.6%
	2	0.0%	0.0%	28.5%	0.0%	4.0%	0.0%	0.0%	3.0%	3.5%	3.1%	0.0%	3.7%	15.4%	4.3%
	3	0.0%	0.0%	0.0%	9.0%	4.4%	0.0%	0.0%	0.0%	0.0%	0.0%	4.7%	0.0%	5.2%	9.0%
	4	3.4%	2.1%	36.0%	18.6%	11.2%	2.1%	6.7%	4.0%	5.5%	1.9%	2.2%	0.0%	47.9%	21.9%
	5	7.4%	0.0%	29.7%	19.7%	8.2%	2.2%	25.0%	0.0%	0.0%	2.2%	15.8%	2.7%	23.6%	9.1%
	6	0.0%	0.0%	8.9%	3.7%	0.0%	0.0%	4.0%	0.0%	0.0%	0.0%	0.0%	0.0%	13.5%	6.2%
	7	0.0%	0.0%	12.2%	3.6%	4.3%	4.3%	4.1%	0.0%	0.0%	3.8%	3.9%	4.2%	10.6%	5.0%
	8	0.0%	0.0%	0.0%	7.5%	7.7%	4.3%	0.0%	0.0%	0.0%	0.0%	6.7%	0.0%	10.3%	9.3%
	9	0.0%	0.0%	9.5%	5.3%	0.0%	0.0%	3.3%	0.0%	7.3%	0.0%	1.4%	0.0%	18.5%	2.8%
	10	4.0%	0.0%	29.2%	6.7%	1.8%	0.0%	1.9%	0.0%	2.1%	0.0%	0.0%	0.0%	17.7%	4.6%
Ped	11	0.0%	0.0%	61.3%	11.3%	4.4%	0.0%	4.4%	1.7%	4.1%	2.0%	0.0%	3.8%	127.3%	8.0%
	12	0.0%	1.3%	10.5%	7.1%	2.2%	0.0%	3.1%	0.9%	18.4%	1.0%	15.7%	0.0%	16.5%	3.8%

*Note*: 1 = CT ABDOMEN PELVIS KIDNEY WO IVCON, 2 = CT ABDOMEN PELVIS W IVCON, 3 = CT ABDOMEN PELVIS WO IVCON, 4 = CT C SPINE WO IVCON, 5 = CT CHEST ABDOMEN PELVIS W IVCON, 6 = CT CHEST PULMONARY ARTERIES W IVCON, 7 = CT CHEST W IVCON, 8 = CT CHEST WO IVCON, 9 = CT HEAD BRAIN WO IVCON, 10 = CT NECK W IVCON, 11 = CT HEAD BRAIN WO IVCON | 00–02, 12 = CT HEAD BRAIN WO IVCON | 15–18.

### 3.4 Diagnostic reference level variability

As shown in Table 5, appreciable variability was observed for the DRL data, with overall CV ranging from 2.8% (adult CT Head Brain WO IVCON) to 21.9% (adult CT C‐Spine WO IVCON) across the study period. The highest annual variability (19.7%) was observed for adult CT Chest Abdomen Pelvis W IVCON in 2021. DRL data were more variable during the 2021–2024 period, followed by a reduction in 2025. Out of the 12 exams evaluated, F‐test results showed significant year‐to‐year changes for 8/12 exams in 2022, decreasing to 6/12 in 2023 and 2/12 in 2024.

## 4 DISCUSSION

Longitudinal evaluation of CT DIR data provides a robust framework for understanding institutional CT dose performance and prioritizing quality assurance efforts. Currently, ACR CT DIR executive summaries provide quarterly assessments of institutional dose performance against national benchmarks. Although this format is operationally simple, it may limit recognition of temporal variability, changes in benchmark values, and exam‐specific patterns in dose performance. The longitudinal approach presented in this study can serve as a complementary analysis that adds value to routine DIR reporting by providing additional insight into institutional dose performance over time.

A central observation of this work is that exceedance behavior was not uniformly distributed across exams when evaluated over time. Rather than widespread marginal exceedance, repeated deviation from DIR benchmarks was concentrated in a small subset of exams, while many exams remained consistently within reference levels across multiple years. This distinction is difficult to appreciate through isolated quarterly review but becomes readily apparent when benchmark relationships are examined longitudinally. Importantly, this supports a prioritization strategy based on persistence and reproducibility rather than isolated threshold crossings.

Evaluation across multiple dose metrics further highlights the importance of longitudinal context. Differences between CTDI_vol_ and DLP behavior demonstrated that reliance on a single metric can lead to incomplete or potentially misleading conclusions. In several exams, including CT Chest W IVCON, CT C‐Spine WO IVCON, and CT Chest Pulmonary Arteries W IVCON, DLP exceedance occurred more frequently than CTDI_vol_ exceedance, emphasizing that examination‐level factors may influence benchmarking outcomes even when scanner output remains relatively stable. Such factors may include differences in scan coverage, inclusion of anatomy beyond protocol‐defined boundaries, or scanner‐specific implementation of examination protocols. Because DLP is directly influenced by scan length, this pattern may help narrow investigations toward review of scan coverage and protocol adherence rather than scanner output parameters alone. Similarly, when available, SSDE provides additional context by accounting for patient size, although incomplete benchmark availability limited its routine use across all exams and pediatric age groups. These observations reinforce that interpretation of dose performance is inherently multi‐dimensional and benefits from coordinated evaluation of complementary metrics over time.

Beyond quantitative exceedance rates, longitudinal visualization provided critical insight into how and when exceedance behavior emerged. Gradual upward drift relative to DIR benchmarks, sustained changes in benchmark position, and abrupt shifts in dose metrics produced distinct longitudinal patterns that could not be distinguished through isolated quarterly review. These patterns may provide additional context regarding the nature of observed dose metric changes and support more focused investigation of potential contributing factors. For example, concurrent increases in CTDI_vol_ and DLP may initially suggest worsening protocol performance; however, if SSDE remains relatively stable over the same period, the observed increase may be attributable to changes in patient size distribution rather than protocol‐related factors. Conversely, persistent increases across all available dose metrics may warrant review of protocol implementation, scanner utilization, or technique parameters. Abrupt changes may instead warrant assessment of examination mapping, data submission integrity, benchmark movement, or registry classification. In this study, CT C‐Spine WO IVCON exam demonstrated abrupt increases in CTDI_vol_ and DLP relative to DIR benchmarks, with SSDE being unavailable. Subsequent detailed review of the exam and submitted data revealed that the reported value was influenced by CT Head Brain acquisitions associated with the CT C‐Spine study, resulting in a registry classification artifact. This produced an apparent dose elevation that did not reflect true CT C‐Spine protocol or technique performance. Such findings demonstrate how longitudinal analysis can help distinguish true opportunities for protocol optimization from changes attributable to benchmark behavior, data misclassification, or other non‐protocol related factors. The ability to differentiate these mechanisms is a key strength of longitudinal analysis and directly addresses the limitations of isolated quarterly review.

While longitudinal visualization provides important context regarding observed dose changes, additional statistical analyses can help quantify and prioritize these findings. Unlike statistical process control (SPC) methods, which are designed to detect unusual variation or process changes over time, the LME framework used in this study quantified exam‐specific deviations from historical performance while accounting for exam‐specific differences within a single model.^13^, ^14^ To support its application as a tool for exam prioritization, the LME analysis used quarterly facility data to characterize longitudinal performance and an indicator for the most recent calendar year (2025) to identify exams demonstrating recent deviations from their historical performance. The annual period was selected to align this assessment with annual CT protocol review, during which exams demonstrating concerning dose performance may be identified for further investigation and potential optimization. In our analysis, the model identified a statistically significant upward deviation in CTDI_vol_ during 2025 and highlighted CT C‐Spine WO IVCON as demonstrating the largest positive shift. Additional exams, including CT Chest W IVCON, CT Abdomen Pelvis W IVCON, and CT Head Brain WO IVCON, also demonstrated upward shifts, illustrating the importance of a systematic framework for evaluating multiple potential optimization targets. While SPC approaches may be effective for identifying when a process has changed, the LME framework provided a quantitative approach for prioritizing where review efforts should be directed.

Complementary variability analysis using the CV and year‐to‐year F‐tests evaluated the consistency of quarterly dose performance over time. While the standard deviation bands provided visual context regarding the absolute temporal variability of DIR benchmark percentiles across the study period, CV provided a normalized measure of relative variability. Evaluating variability in both the facility median and DRL provides additional context regarding the stability of the two quantities used in benchmark comparison. Importantly, variability is not itself an indicator of elevated or acceptable dose performance; an exam may demonstrate stable quarterly performance while still exhibiting elevated dose metrics. Rather, increased or changing variability may provide an additional signal for further investigation when interpreted alongside longitudinal trends and benchmark comparisons. This was demonstrated by CT C‐Spine WO IVCON, where high variability, together with its longitudinal trend and LME findings, provided converging evidence that further investigation was warranted. From a quality assurance perspective, such variability may warrant review of factors contributing to reduced consistency in dose performance, while persistently low variability may provide confidence that exam performance remains stable over time.

By emphasizing sustained, reproducible patterns rather than isolated outliers, longitudinal analysis improves confidence in deciding when an exam warrants investigation, focuses limited quality assurance resources where they are most likely to yield benefit, and reduces alert fatigue. It further facilitates multidisciplinary communication and supports continuous quality improvement by documenting performance over time and providing defensible context for accreditation or internal review. It is important to note that this approach does not replace existing DIR reporting but rather builds upon it, transforming routinely available registry outputs into a more informative and actionable tool.

Despite the utility of this analysis, several practical considerations should be acknowledged. Pediatric DIR evaluation introduced challenges related to age stratification, reporting volume, and benchmark availability. Because benchmark data are reported separately by exam‐age group combinations, the number of evaluable reporting intervals varied substantially across age groups, and SSDE benchmark data were frequently unavailable, limiting comprehensive cross‐metric evaluation. While repeated exceedance patterns were observed in select pediatric exam‐age groups, the varying number of evaluable reporting intervals and incomplete benchmark availability require cautious interpretation of pediatric longitudinal trends. The year‐to‐year F‐test analysis was also limited by the number of quarterly observations available within each calendar year (*n* = 4), which reduces statistical power to detect differences in variance, particularly when differences are modest. F‐test results should therefore be interpreted alongside the quantitative CV values and longitudinal trends when evaluating changes in dose variability. In general, this study reflects data from a single institution and may therefore be influenced by local scanner configuration, protocol mapping practices, and clinical workflow. Additionally, the exceedance definition applied in this study was intentionally conservative and focused on facility median behavior relative to DIR benchmarks. Alternative exceedance definitions or threshold criteria may yield different sensitivity to short‐term variation and could be a topic of future investigation. Future work may also focus on integrating longitudinal analysis with protocol‐level parameter evaluation. Once exams requiring further review have been identified and evidence suggests a protocol‐related issue, techniques such as kernel density estimation (KDE) may help identify the specific parameters contributing to observed dose behavior and guide targeted optimization efforts.

## 5 CONCLUSION

Longitudinal analysis of ACR DIR executive summary data offers a meaningful extension beyond isolated quarterly review by characterizing exceedance behavior over time, accounting for benchmark variability, and distinguishing persistent exam behavior from transient changes. Using exceedance rates, longitudinal trends, and complementary statistical analyses, this framework improves interpretation of institutional CT dose performance, enables targeted exam triage, and provides a defensible structure for ongoing quality assurance and accreditation support. As participation in DIR continues to expand, longitudinal review represents a practical and scalable approach for institutions seeking to derive greater value from registry‐based dose benchmarking.

## AUTHOR CONTRIBUTIONS

**Azmul H. Siddique**: Conceptualization; methodology; data curation; formal analysis; investigation; visualization; writing—original draft; writing—review & editing. **Mike Weaver**: writing—review & editing. **Gary Ge**: writing—review & editing. **Leslie Anaskevich**: Resources. **Jie Zhang**: Supervision; conceptualization; writing—review & editing. All the authors have read and approved the final version of the manuscript.

## CONFLICT OF INTEREST STATEMENT

The authors declare no conflicts of interest.

## ETHICS AND IRB COMPLIANCE STATEMENT

This study utilized de‐identified, aggregated CT dose data obtained from the American College of Radiology Dose Index Registry (DIR) through the National Radiology Data Registry. In accordance with institutional policies, this work did not meet the definition of human subject's research and did not require Institutional Review Board review.

## Data Availability

The data supporting the findings of this study are available from the corresponding author upon reasonable request. Authors will share data upon request to the corresponding author.
